# Dysphagia in head and neck cancer patients following intensity modulated radiotherapy (IMRT)

**DOI:** 10.1186/1748-717X-6-1

**Published:** 2011-01-05

**Authors:** Evangelia Peponi, Christoph Glanzmann, Bettina Willi, Gerhard Huber, Gabriela Studer

**Affiliations:** 1 Department of Radiation Oncology, University Hospital Zurich, Zurich, Switzerland; 2 Department of Pediatrics, Civic Hospital of Lugano, Lugano, Switzerland; 3 Department of Otorhinolaryngology, Head and Neck Surgery, University Hospital Zurich, Zurich, Switzerland

## Abstract

**Background:**

To evaluate the objective and subjective long term swallowing function, and to relate dysphagia to the radiation dose delivered to the critical anatomical structures in head and neck cancer patients treated with intensity modulated radiation therapy (IMRT, +/- chemotherapy), using a midline protection contour (below hyoid, ~level of vertebra 2/3).

**Methods:**

82 patients with stage III/IV squamous cell carcinoma of the larynx, oropharynx, or hypopharynx, who underwent successful definitive (n = 63, mean dose 68.9Gy) or postoperative (n = 19, mean dose 64.2Gy) simultaneous integrated boost (SIB) -IMRT either alone or in combination with chemotherapy (85%) with curative intent between January 2002 and November 2005, were evaluated retrospectively. 13/63 definitively irradiated patients (21%) presented with a total gross tumor volume (tGTV) >70cc (82-173cc; mean 106cc). In all patients, a laryngo-pharyngeal midline sparing contour outside of the PTV was drawn. Dysphagia was graded according subjective patient-reported and objective observer-assessed instruments. All patients were re-assessed 12 months later. Dose distribution to the swallowing structures was calculated.

**Results:**

At the re-assessment, 32-month mean post treatment follow-up (range 16-60), grade 3/4 objective toxicity was assessed in 10%. At the 32-month evaluation as well as at the last follow up assessment mean 50 months (16-85) post-treatment, persisting swallowing dysfunction grade 3 was subjectively and objectively observed in 1 patient (1%). The 5-year local control rate of the cohort was 75%; no medial marginal failures were observed.

**Conclusions:**

Our results show that sparing the swallowing structures by IMRT seems effective and relatively safe in terms of avoidance of persistent grade 3/4 late dysphagia and local disease control.

## Background

Limited data are available on the long term swallowing function in intensity modulated radiotherapy (IMRT) treated patients at risk for dysphagia [1-3].

We aimed to evaluate the objective and subjective long term swallowing function, and to relate dysphagia to the radiation dose delivered to the critical anatomical structures in our consecutively IMRT (+/- chemotherapy) treated head and neck cancer patients.

We focused on serious subjective as well as objective symptoms (grade 3/4 late effects).

## Methods

### Patient, disease and staging characteristics

A total of 82 out of 96 eligible patients 'at risk' for dysphagia due to a stage III/IV squamous cell carcinoma of the larynx, oropharynx or hypopharynx agreed to participate in our retrospective assessment. All included patients were successfully treated with curative intent by simultaneous integrated boost (SIB)-IMRT either alone or in combination with chemotherapy or surgery at our department between January 2002 and November 2005. Seventy patients (85%) received concurrent cisplatin chemotherapy (40mg/m2 i. v. weekly).

Exclusion criteria included loco-regional recurrence at the time of assessment of swallowing dysfunction, a follow-up period <4 months at the first assessment, patients having tracheostomy tubes and/or laryngectomy, and loco-regional tumor stage ≤T1/2 N0.

Analysis has been performed after institutional research ethics board approval. First, EORTC questionnaires regarding quality-of-life (QOL) and SOMA LENT scale regarding late toxicity accompanied with an informed consent form were mailed out to the patients, who were already informed by phone. The subjective answers resulted from a first assessment (mean 20 months; range: 4-40 months), based on a questionnaire for each patient. All patients -with special consideration to those presenting with late toxicity > grade 2- have been re-assessed objectively one year later (mean 32 months, range 16-60). The 5 year local disease control and dysphagia grade 3/4 rates were based on the most recent follow up assessment ('last time seen').

Included in this analysis were 19 consecutive eligible patients treated in the indicated time period, who underwent surgery (without tracheostomy or laryngectomy) followed by postoperative IMRT, as the postoperative set up was considered similarly 'risky' for the development of late term dysphagia (fibrosis, edema), and of additional informative value.

In addition, one interesting case of a patient who underwent contra-lateral cobalt irradiation 30 years ago was also included. This patient with a T3N2b lateral oropharynx cancer experienced grade 4 dysphagia at the subjective assessment. She received total IMRT dose of 69.6Gy unilaterally (daily dose: 2.11Gy) and 5 cycles of concurrent cisplatin, after having been irradiated 30 years ago to the contra-lateral neck and tonsil with a total dose of 60Gy by a Co^60^; the cumulative dose received by the swallowing structures could not be estimated. Esophagus dilatations achieved temporary results; however, although she remains PEG dependent, she is able to swallow her saliva, and remained disease free at the 4-year follow-up visit.

All patients were staged using the 2002 American Joint Committee on Cancer (AJCC) criteria [4]. Patient and disease characteristics are listed in Table 1. Mean age of the cohort was 61 years (range 34-80). Volumetric staging is shown in Table 2.

**Table 1:** Patient and disease characteristics (n = 82)

Characteristics	No of patients	%
**Gender**		
Male	68	83
Female	14	17
		
**Primary site**		
Oropharynx central	26	32
Oropharynx lateral	29	35
Hypopharynx	18	22
Larynx	9	11
		
**Stage III/IV**	82	100
		
**RT intention**		
primary	63	77
postoperative	19	23
		
**Concomitant CT**	70	85
≥4 cycles	63	77
		
**previous RT**	1	1

**Abbreviations: **No: number, RT: Radiotherapy. CT: Chemotherapy.

**Table 2:** Volumetric staging in patients treated with primary radiotherapy (n = 63)

	total gross tumor volume (tGTV)			
	
Primary site	mean (range)	1-15cc	16-70cc	>70cc
**Oropharynx**				
base of tongue	34cc (9-127cc)	2	13	3
tonsil/lateral mesopharynx	51cc (4-171cc)	1	11	7
				
**Larynx**				
Glottis	12cc (1-29cc)	1	1	0
Supraglottis	21cc (6-54cc)	2	5	0
				
**Hypopharynx**	46cc (5-173cc)	3	11	3

**Total**	**44cc (1-173cc)**	**9 (14%)**	**41 (65%)**	**13 (21%)**

### Evaluation and scoring of late toxicity

Normal tissue effects were graded according to the Radiation Therapy Oncology Group (RTOG)/European Organization for Research and Treatment of Cancer (EORTC) radiation morbidity scoring criteria [5].

Swallowing dysfunction and dysphagia were additionally graded with subjective patient-reported and objective observer-assessed instruments. Patient-reported clinical swallowing function was evaluated using the "European Organization for Research and Treatment of Cancer (EORTC) head-and-neck 35-item swallowing and aspiration (QLQ-H&N35)" quality-of-life (QOL) questionnaire.

Observer-assessed dysphagia was assessed according to the SOMA LENT scale for head-and-neck carcinoma radiotherapy objective criteria (German Version). During the course of irradiation, all patients were clinically assessed at regular weekly intervals, and 2 weeks to 2 months after completion of treatment. Four to 6 weeks after completion of IMRT, all patients were also seen regularly in our joint clinic at the Department of Head and Neck Surgery. Institutional standards for patient assessment included physical examination with additional flexible fiberoptic endoscopy at the Department of Head and Neck Surgery approximately every 2 months in the first year of follow-up, every 3 months in the second to third year and every 6 months in the fourth to fifth year.

### Treatment characteristics

Patients were immobilized from head to shoulders with commercially available thermoplastic masks in the supine position. CT images (2 mm slice thickness) were acquired from the top of the vertex to the level of the carina with contrast agent infusion in non-operated patients.

We used an extended-field IMRT (EF-IMRT) technique, where the primary tumor was treated in one phase along with the regional lymph nodes. Irradiation was delivered with five or seven coplanar beam angles by a 6-MV dynamic MLC system (sliding window technique) (Varian Medical Systems, CA).

As previously described [1] an accelerated SIB- IMRT technique was performed with a daily dose of 2.00-2.35Gy (total dose: 63-75Gy) to the primary tumor and positive neck nodes in the definitive RT cases (n = 63) and a daily dose of 1.80-2.00Gy to a total dose of 60-66Gy in postoperative cases (n = 19). For intensity optimization the prescribed dose should encompass at least 95% of the PTV. Additionally, no more than 20% of any PTV would receive >110% of its prescribed dose, while no more than 1% of any PTV would receive <93% of the desired dose. The mean total treatment time was 45.3 days (32-55 days).

**Figure 1:**
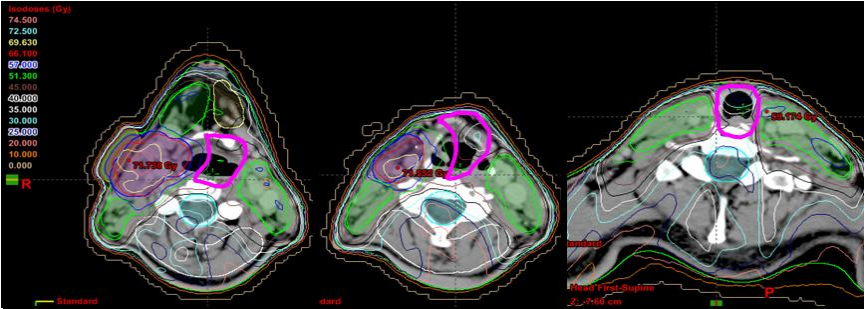
**Example: midline shielding as used according to our internal IMRT guidelines (pink contour, below hyoid/C3)**.

The protection of anatomical swallowing structures was routinely performed by drawing a laryngo-pharyngeal midline 'shielding' contour outside the PTVs in all cases. This sparing structure has been defined prospectively in January 2002, when we implemented IMRT clinically, and was provided to be used in all midline areas where no PTV was required. This structure may include esophageal, laryngeal, and pharyngeal structures. Aimed dose constraint for this midline shielding was a mean dose (Dmean) below 45Gy (Figure 1).

In oropharyngeal cancer patients, this structure was usually contoured from the level of the hyoid (below the lateral retropharyngeal lymph nodes, corresponding ~to the cervical vertebra 2/3, Figure 1) to the lowest level at which PTVs were drawn. In hypopharyngeal cancer patients, midline protection is often limited to some aspects of the larynx to just prevent laryngeal structures from full tumor dose.

### Clinical factors

The clinical variables examined for correlation with grade 3-4 late toxicity included age, gender, primary site, tumor stage, tumor volume, therapy sequence, addition of systemic therapy and IMRT treatment schedules.

### Dosimetric factors

The dose distribution to the swallowing structures was calculated on the original IMRT treatment plans. Based on studies published so far [6-9], and with regard to the swallowing apparatus, the following anatomic structures were retrospectively identified and delineated on the axial CT-slices of each plan: the pharyngeal constrictor muscles (PCs) - superior, middle and inferior-, the glottic and supraglottic larynx (GSL) and the muscular compartment of the esophagus inlet (eim). In brief, the superior constrictor muscle (scm) was defined from the caudal tips of the pterygoid plates through the upper edge of the hyoid bone, the middle constrictor muscle (mcm) was defined from the upper through the lower edge of the hyoid and the inferior constrictor muscle (icm) was defined from below the hyoid through the inferior edge of the cricoid. A structure named PCs was outlined to involve the constrictors as a single structure. The larynx (GSL) was contoured from the tip of the epiglottis superiorly to the bottom of the cricoid inferiorly. Caudal to the inferior border of the cricoid, the esophagus (eim) was contoured, with its caudal-most extent corresponding to the caudal-most extent of the low neck target volumes. Dose-volume histograms to the swallowing structures were assessed and mean dose, maximum point dose (Dmax), minimum point dose (Dmin), V_30 _(volume of a structure receiving >30Gy), V_50 _(volume of a structure receiving >50Gy), V_60 _(volume of a structure receiving >60Gy), V_65 _(volume of a structure receiving >65Gy), V_70 _(volume of a structure receiving >70Gy), D_50 _(dose received by 50% volume of a structure/median dose) and D_80 _(dose received by 80% volume of a structure) were calculated.

### Statistical analysis

Statistical calculations of Kaplan Meier curves were performed using StatView^® ^program (Abacus Concepts Inc., Berkeley, CA). A *p *value of ≤0.05 was considered statistically significant.

## Results

Between January 2002 and November 2005, a total of 82 out of 96 eligible patients successfully treated with SIB- IMRT agreed to participate in our study. 80 patients responded to all given questionnaires; 2/82 patients declined to return the questionnaires, however, agreed to allow using their objective data as assessable from regular follow up visits. One previously irradiated patient was excluded from the dysphagia analysis.

### Late toxicity

At the first post-treatment follow-up (mean 20 month, range 4-48), any subjective grade 3/4 toxicity (G3/4) was reported by 14/80 patients (18%), while 66/80 patients (82%) experienced grade 0-2 toxicity. At the second follow up (objective assessment), grade 3/4 toxicity rate was 10% (8/78) (two patients excluded because of tumor recurrence, two patients lost to follow-up); Table 3 shows subjective and objective late toxicity. The mean dose and dose range in definitively irradiated versus postoperatively irradiated patients is indicated in Table 1.

**Table 3:** Frequency of grade 3/4 (G3/4) late toxicity at the subjective (mean 20 months; range 4-40) and objective (mean 32 months; range 16-60) assessment

	Grade 3/4 late term toxicity		
	
Parameter	S u b j e c t i v e		O b j e c t i v e
Swallow pain	**1**		**na**
			
Dysphagia	**2**	(1 definitive/	**2 **(same pts. as subjective)
		1postoperative)	
Taste alteration	**9**		**na**
			
Xerostomia	**3**		**6**
			(same 3 pts as subjective + 3 others)
Weight loss ≥10%	**na**		**0**
			
Hoarseness	**0**		**0**

**Total No of pts**	**14/80 (18%)**		**8/78 (10%)**

**Abbreviations: **na: not assessable, pts: patients, No: number

### **Prevalence of long term dysphagia **(re-irradiated patient excluded)

At the patient reported first assessment (mean 20 months, range 4-40), 77/79 patients experienced dysphagia grade 0-2; five patients (8%) experienced dysphagia grade 2, symptom that continued to persist only in one patient by reevaluation (objective assessment, mean 32 months, range 16-60). Persistent dysphagia grade 3/4 was found in one patient (1%). There were no cases of clinically symptomatic pneumonia as a potential consequence of aspiration reported by patients or stated in the patient charts (no radiological swallowing tests performed).

**Figure 2:**
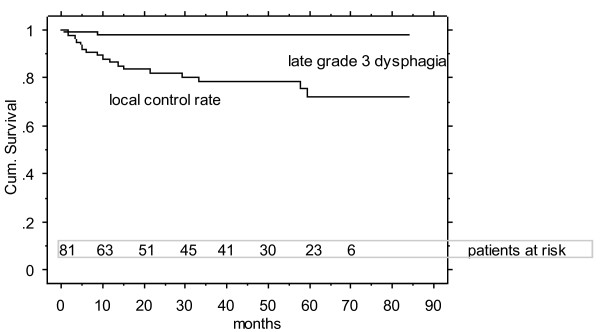
**5-year local control rate (75%) and rate of freedom of grade 3/4 late dysphagia (re-irradiated patient excluded, 99%)**.

At the second evaluation (mean 32 month post treatment; n loco-regionally controlled patients with no previous radiation = 77) as well as at the most recent follow up ("patient last seen", mean 50 months, range 16-85, Figure 2), persisting swallowing dysfunction grade (2-) 3 was subjectively and objectively assessed by 1 patient.

### Weight loss/PEGs

Percutaneous endoscopic gastrostomy feeding tubes (PEGs) were placed before or during treatment in 21 of 82 patients (26%). The mean time to PEG tube removal was 8 months (range 5-25). At the time of the first analysis (20-month follow up), 6/21 patients (7% of all, ~1/3 of the PEG patients) were still using PEG for some or all of their nutrition. Patients sustained median weight loss of 5.1 kg (range 0-20 kg) during treatment, while one year post treatment there was no patient who had lost >10% of body weight. Only two of those 6 patients remained PEG-dependent (10% of all PEG patients, 2% of the entire cohort); the other 4 patients regained independence of PEG 14, 16, 33 and 36 months after completion of IMRT, respectively. In none of the patients who remained loco-regionally disease free, a PEG had to be placed during the monitored follow up period/replaced once the PEG has been removed.

### Swallowing structure doses

The median doses (median of the median dose) to the swallowing structures, the partial volumes receiving specified doses (V_D_) in all patients and comparable results of reported series are detailed in Tables 4 and 5.

**Table 4:** Median doses (median of the median dose) to the swallowing structures in all patients and comparison to reported series

Swallowing structures		Current study		**Feng et al 2007 **[21]		Levendag et al* 2007 [7]	
		
		Median	Range	Median	Range	Median	Range
**PCs**	Volume (cc)	20	11-32	NA	NA	NA	NA
	Dose (Gy)	59	39-66	64	51-72	48	6.0-73.0

**scm**	Volume (cc)	12	6-21	NA	NA	NA	NA
	Dose (Gy)	59.4	24-69	68	57-74	51	22.0-73.0

**mcm**	Volume (cc)	3.6	1.5-9	NA	NA	NA	NA
	Dose (Gy)	59	37.0-71.5	64	53-75	48	11.0-72.0

**icm**	Volume (cc)	3.7	0.9-7	NA	NA	NA	NA
	Dose (Gy)	53	29-73	51	30-70	32	6.0-73.0

**GSL**	Volume (cc)	15.3	6.4-23	NA	NA	NA	NA
	Dose (Gy)	53	28-70	55	22-72	NA	NA

**eim**	Volume (cc)	5.4	0.7-44	NA	NA	NA	NA
	Dose (Gy)	39	16.0-67.8	44	15-66	18	3.0-64.0

*The results are not entirely comparable with the study of Levendag et al [7]**, **as there is a difference in the delineation of muscular structures. In Levendag et al. the anterior part of scm and mcm were not delineated and eim was defined as the proximal 1 cm of the esophageal inlet, regardless of the caudal-most extent of the low neck target volumes.

**Abbreviations: **PCs: pharyngeal constrictor muscles, scm: superior constrictor muscle, mcm: middle constrictor muscle, icm: inferior constrictor muscle, GSL: glottic and supraglottic larynx, eim: muscular compartment of the esophagus inlet, NA: not assessed

**Table 5:** Partial volumes receiving specified doses (VD) to the swallowing structures in all patients and comparison to the data as reported by Feng et al 2007 21

	PCs, median (range)				GSL, median (range)				eim, median (range)		
	
	V50 (%)	V60 (%)	V65 (%)	V70 (%)	V50 (%)	V60 (%)	V65 (%)	V70 (%)	V50 (%)	V60 (%)	V65 (%)
**Current study**	88.5 (10-100)	43.9 (0-94)	29 (0-60)	7.3 (0-40)	3.9 (1-100)	21.1 (0-98)	8.9 (0-94)	0 (0-67)	5.1 (0-100)	0 (0-87)	0 (0-84)
**Feng et al **[21]	90 (58-100)	73 (36-100)	57 (20-99)	NA	69 (1-100)	37 (0-100)	20 (0-100)	NA	14 (0-100)	0 (0-100)	0 (0-78)

**Abbreviations: **PCs: *pharyngeal constrictor muscles*, GSL: *glottic and supraglottic larynx*, eim: *muscular compartment of the esophagus inlet*, NA: *not assessed*.

### Long term local control and overall survival

The 3 and 5 year local control rates of the assessed cohort were 78 and 75% (Figure 2), the corresponding overall survival rates were 80 and 77%, respectively (Kaplan Meier survival curves, December 2010). None of the local failures were found related to the midline protection structure (all failures analysed: no medial marginal failures).

## Discussion

Recent gains in the management of head and neck cancer have been achieved due to concurrent chemo-radiotherapy with altered fractionated three-dimensional conformal radiotherapy (3D-CRT) or IMRT technique [10-12]. The use of these high intensity treatments has resulted in considerable rates of swallowing dysfunction, both acute (15-63%) and long term (3-21%) [13-20]. Comprehensive data on late toxicity from randomized and nonrandomized trials, however, are sparse.

In our cohort of patients treated with SIB-IMRT either alone or in combination with chemotherapy or surgery, the rate of grade 3/4 long term dysphagia was 1%, comparable to that seen in other IMRT studies, and considerably better than that observed in 3D-CRT studies (Table 6). These findings of a low rate of severe dysphagia in a patient cohort at risk motivate efforts to reduce the doses to the swallowing structures, fact which could reduce the severity and prevalence of dysphagia. This may be reached by a simple protection structure along the midline were no PTV is needed (Figure 1). Analysis of the relationship between the swallowing structure doses and the development of late dysphagia were limited due to the single event, precluded statistical significance. Median doses of the swallowing structures in the own cohort were comparable to reported series [7,21] (Table 4, 5). The limitation of our study was the retrospective nature of the analysis, whereas midline protection contouring (Figure 1) was prospectively performed as part of our internal IMRT guidelines. Similar to the parotid gland protection, no oncological compromises are acceptable in contouring the midline sparing structure. The group of Eisbruch et al [2] suggested that the high loco-regional control rates have not been compromised by the efforts to spare the parts of the swallowing structures not involved by tumor and not at risk of subclinical disease. In addition, in a previous evaluation of our hypopharynx-larynx patient cohort [22] treated with IMRT using midline sparing as far as feasible, local failures were not found related to the midline sparing structures.

**Table 6:** Results from selected series regarding late toxicity in head and neck cancer patients treated with RT ± chemotherapy

Technique	Authors [reference]	year	No of patients	median follow up (months)	stage lll/lV (%)	Chemotherapy (%)	grade 3/4 late toxicity
	Chao et al [25]	2003	74	30	93	23	0
I							
	de Arruda et al [14]	2006	50	18	92	86	3 (6%)
M							
	Lee et al [17]	2006	41	31	100	100	5 (12%)
R							
	Studer et al [1]	2006	115	18	52	78	18 (15%)
T							
	present study	2010	81*	55	100	85	7 (9%)
	Denis et al [26]	2003	44	60	100	61	30 (68%)
3D-CRT							
	Huguenin et al [19]	2004	224	39	97	50	G3: 92 (41%)
							G4: 5 ("%)

**Abbreviations: **IMRT: Intensity modulated radiation therapy; 3D-CRT: three-dimensional conformal radiotherapy (*patient with previous Cobalt radiation therapy excluded)

The low percentage of PEG tube dependence (7%) at the mean 20-month follow-up may be interpreted as a surrogate of limited swallowing problems.

Published analyses focused on predicting the probability of severe acute or late dysphagia during or after RT [23,24] between patient-rated and objective assessment of dysphagia are conflicting.

Patients' satisfaction with their swallowing function, in addition with the objective parameters 'body weight' and 'dependency of a long term PEG', are reliable answer and were found congruent with the objective grading. No specific tests were performed to detect potentially aspiration-related, clinically not obvious pneumonia.

## Conclusions

In conclusion, IMRT using a midline contour to spare swallowing structures outside PTVs is relatively safe and effective in terms of local disease control and avoidance of persistent late dysphagia. The subjective patients' estimation of late dysphagia was compatible with the objective assessment of swallowing dysfunction.

## Competing interests

The authors declare that they have no competing interests.

## Authors' contributions

GS and CG conceived of the study, carried out its design and supervised the coordination. BW performed all phone call interviews with patients, sent out and analysed the QoL questionnaire forms. EP carried out the specific contouring work, analysed the related DVHs, and drafted the manuscript. GH was mainly involved/in charge with the clinical post treatment follow up visits of all patients. All authors read and approved the final manuscript.
